# The spectrum of *EWSR1*-rearranged neoplasms at a tertiary sarcoma centre; assessing 772 tumour specimens and the value of current ancillary molecular diagnostic modalities

**DOI:** 10.1038/bjc.2017.4

**Published:** 2017-01-31

**Authors:** Jonathan Noujaim, Robin L Jones, John Swansbury, David Gonzalez, Charlotte Benson, Ian Judson, Cyril Fisher, Khin Thway

**Affiliations:** 1 Sarcoma Unit, Royal Marsden Hospital, London SW3 6JJ, UK; 2 Clinical Cytogenetics, Royal Marsden Hospital, Surrey SM2 5NG, UK; 3 Molecular Diagnostics, Royal Marsden Hospital, Surrey SM2 5NG, UK

**Keywords:** *EWSR1*, Ewing sarcoma, fluorescence *in situ* hybridisation, fusion transcripts, gene rearrangement, molecular genetics, translocation, reverse transcription–PCR

## Abstract

**Background:**

*EWSR1* rearrangements were first identified in Ewing sarcoma, but the spectrum of *EWSR1*-rearranged neoplasms now includes many soft tissue tumour subtypes including desmoplastic small round cell tumour (DSRCT), myxoid liposarcoma (MLPS), extraskeletal myxoid chondrosarcoma (EMC), angiomatoid fibrous histiocytoma (AFH), clear cell sarcoma (CCS) and myoepithelial neoplasms. We analysed the spectrum of *EWSR1*-rearranged soft tissue neoplasms at our tertiary sarcoma centre, by assessing ancillary molecular diagnostic modalities identifying *EWSR1*-rearranged tumours and reviewing the results in light of our current knowledge of these and other Ewing sarcoma-like neoplasms.

**Methods:**

We retrospectively analysed all specimens tested for *EWSR1* rearrangements by fluorescence *in situ* hybridisation (FISH) and/or reverse transcription–PCR (RT–PCR) over a 7-year period.

**Results:**

There was a total of 772 specimens. FISH was performed more often than RT–PCR (*n*=753, 97.5% *vs n*=445, 57.6%). In total, 210 (27.9%) specimens were FISH-positive for *EWSR1* rearrangement compared to 111 (14.4%) that showed *EWSR1* fusion transcripts with RT–PCR. Failure rates for FISH and RT–PCR were 2.5% and 18.0%. Of 109 round cell tumours with pathology consistent with Ewing sarcoma, 15 (13.8 %) cases were FISH-positive without an identifiable *EWSR1* fusion transcript, 4 (3.7%) were FISH-negative but RT–PCR positive and 4 (3.7%) were negative for both. FISH positivity for DSRCT, MLPS, EMC, AFH and CCS was 86.3%, 4.3%, 58.5%, 60.0% and 87.9%, respectively. A positive FISH result led to diagnostic change in 40 (19.0%) *EWSR1*-rearranged cases. 13 FISH-positive cases remained unclassifiable.

**Conclusions:**

FISH is more sensitive for identifying *EWSR1* rearrangements than RT–PCR. However, there can be significant morphologic and immunohistochemical overlap between groups of *EWSR1-*rearranged neoplasms, with important prognostic and therapeutic implications. FISH and RT–PCR should be used as complementary modalities in diagnosing *EWSR1*-rearranged neoplasms, but as tumour groups harbouring *EWSR1* rearrangements are increasingly characterised and because given translocations involving *EWSR1* and its partner genes are not always specific for tumour types, it is critical that these are evaluated by specialist soft tissue surgical pathologists noting the morphologic and immunohistochemical context. As RT–PCR using commercial primers is limited to only the most prevalent *EWSR1* fusion transcripts, the incorporation of high-throughput sequencing technologies into the standard diagnostic repertoire to assess for multiple molecular abnormalities of soft tissue tumours in parallel (including detection of newly characterised Ewing sarcoma-like tumours) might be the most effective and efficient means of ancillary diagnosis in future.

Soft tissue neoplasms are a heterogeneous group unified only by their differentiation towards various mesenchymal lineages. Their classification is incomplete and continues to be refined, aided particularly by several recent molecular advances. A proportion of soft tissue tumours harbours characteristic, reproducible genetic abnormalities, including chromosomal translocations that result in the fusion of two separate genes, of which there are almost 100 uniquely identified in sarcoma (Mertens and Tayebwa, 2014); molecular techniques are therefore a crucial and routine adjunct to diagnosis. First observed to be rearranged in Ewing sarcoma (Aurias *et al*, 1983), the Ewing sarcoma breakpoint region 1 gene (*EWSR1*) on chromosome 22q12 encodes a 656 amino acid nuclear protein including a carboxy-terminus 87-amino acid RNA-binding domain (exons 11–13) involved in protein–RNA binding, transcription and RNA metabolism (Fisher, 2014). Its role in cancer cell progression is still unclear, although it may play a critical role in DNA damage response and cell division (Li *et al*, 2007; Paronetto, 2013). While initially thought specific for Ewing sarcoma (formerly the Ewing sarcoma/primitive peripheral neuroectodermal tumour (PNET) family of tumours) (Dockhorn Dworniczak *et al*, 1994), characteristic rearrangements between *EWSR1* and partner genes have been documented in both tumours of mesenchymal and non-mesenchymal lineage, including desmoplastic small round cell tumour (DSRCT; Ladanyi and Gerald, 1994; Antonescu *et al*, 1998), myxoid liposarcoma (MLPS; Panagopoulos *et al*, 1996; Dal Cin *et al*, 1997; Hosaka *et al*, 2002), extraskeletal myxoid chondrosarcoma (EMC; Sciot *et al*, 1995; Clark *et al*, 1996), angiomatoid fibrous histiocytoma (AFH; Hallor *et al*, 2005; Rossi *et al*, 2007; Thway and Fisher, 2015; Thway *et al*, 2015b), clear cell sarcoma of soft tissue (CCS; Hisaoka *et al*, 2008; Wang *et al*, 2009) and clear cell sarcoma-like tumours of the gastrointestinal tract (CCSLGT; Thway and Fisher, 2012; Wang and Thway, 2015), primary pulmonary myxoid sarcoma (PPMS; Thway *et al*, 2011), myoepithelial tumours of skin, soft tissue and bone (Antonescu *et al*, 2010a; Antonescu *et al*, 2010b; Thway and Fisher, 2014; Thway *et al*, 2015a), and more rarely in low-grade fibromyxoid sarcoma (LGFMS; Lau *et al*, 2013) and sclerosing epithelioid fibrosarcoma (SEF; Doyle *et al*, 2012; Arbajian *et al*, 2014). *EWSR1* rearrangements can be easily detected in the routine setting by fluorescence *in situ* hybridisation (FISH) with break-apart probes, and corresponding fusion transcripts by reverse transcription–PCR (RT–PCR) studies, usually using commercial probes and primers respectively. In view of the increasing prominence of *EWSR1* rearrangement in soft tissue neoplasms, we evaluated the utility of FISH and RT–PCR as ancillary diagnostic tools in assessing potential *EWSR1*-rearranged neoplasms in today's current practice, and assessed the spectrum of *EWSR1*-rearranged neoplasms at our tertiary centre over the course of the establishment of the ancillary molecular diagnostics and molecular cytogenetics services.

## Materials and methods

All neoplasms which had FISH and/or RT–PCR performed to assess for *EWSR1* rearrangement or for fusion transcripts containing *EWSR1* were identified from 2008 to 2015 from the prospectively maintained Royal Marsden Hospital (RMH) molecular genetics and cytogenetics databases (DG and JS). Information regarding diagnoses, morphology and immunohistochemistry was found on the matched surgical pathology reports. Prior to *EWSR1* rearrangement testing, diagnoses were made from morphology and immunohistochemistry by one or two (KT and CF) soft tissue specialist (consultant/attending) pathologists. Tumours with pathologic features in keeping with an *EWSR1*-rearranged neoplasm were tested to confirm diagnosis, as the presence of *EWSR1* fusion transcripts, or of the presence of an *EWSR1* rearrangement in the appropriate pathologic and clinical context, would represent the diagnostic gold standard; tumours with pathologic features suggestive of an *EWSR1*-rearranged neoplasm or in which an *EWSR1*-rearranged neoplasm could not be excluded were also tested. Finally, tumours that were difficult to classify morphologically and immunohistochemically that could conceivably represent atypical variants of *EWSR1*-rearranged neoplasms were also tested. FISH or RT–PCR tests were requested only by a consultant/attending soft tissue pathologist, and chosen in light of the clinical and pathologic picture and tissue availability. Further diagnostic interpretation was then made according to the results of ancillary molecular testing. All cases were formalin-fixed and paraffin-embedded (FFPE) and included both core biopsy and excision specimens of material biopsied or resected at our centre, or were external cases that had been sent for review or second opinion.

For FISH, 2- to 4-*μ*m thick FFPE sections were dewaxed overnight at 60 °C, treated with hot buffer wash at 80 °C (2–3 h) then proteolytic enzyme treatment at 37 °C, and finally washed in distilled water and then an alcohol series before addition of an *EWSR1* break-apart probe (Vysis, Abbott Laboratories Ltd, Maidenhead, UK). Hybridisation was performed overnight according to the manufacturer's protocols. An extra pressure cooker step was more recently added, whereby 35 ml of antigen retrieval buffer and 3.5 l of distilled water were brought to a boil in a pressure cooker, the dewaxed slides added, the pressure raised and the cooker cooled after five minutes, after which the slides were washed twice in distilled water before proceeding to the hybridisation stage (Vroobel *et al*, 2016). After FISH, images of the sections were captured using a cooled charged-coupled device camera. To minimise nuclear truncation artefacts, only nuclei with at least two *EWSR1* signals were evaluated. Overlapping tumour nuclei were also excluded from evaluation to decrease false-positive scoring. With this probe used in this study, separated signals that were at least three signal widths apart were scored as positive. However, occasional, scattered positive cells are not sufficient for a case to be classed as positive; laboratory policy is that there should be at least 5% of evaluable nuclei in at least three high-power fields, excluding fields with no positive cells present. Overall, a FISH study was classed as positive if two analysts were in agreement that the number of nuclei with clearly separated signals was sufficient without needing a formal count. In very rare cases, a very small number of abnormal cells was seen and then a third, usually more senior analyst would give an assessment and a formal count would be made. If the proportion of typically abnormal cells in three selected high-power fields constituted less than 5% of the assessable cells, then this would be either disregarded or it would be reported with a caveat to say that there were too few cells to support the diagnosis.

For reverse transcription real-time quantitative–PCR(RQ–PCR), RNA was extracted from FFPE samples using the RecoverAll Total Nucleic Acid Extraction kit (Ambion Ltd., Cambridgeshire, UK) and transcribed into complementary DNA (cDNA) using the High Capacity cDNA kit (Applied Biosystems, Warrington, UK) according to the manufacturers' recommendations. Amplification of *B2M* was performed to assess the quality of the RNA as described previously (Thway *et al*, 2016). RQ–PCR reactions were performed to assess for any or all of the following fusion transcripts: *EWSR1-NR4A3, TAF15-NR4A3, EWSR1-FLI1, EWSR1-ERG, EWSR1-WT1, EWSR1-ATF1* and *EWSR1-CREB1*). Primers and probe sequences were adapted from the literature (Okamoto *et al*, 2001; Antonescu *et al*, 2002; Jin *et al*, 2003; Hostein *et al*, 2004; Coindre *et al*, 2006; Antonescu *et al*, 2007; Lewis *et al*, 2007) (please see Supplementary Table A for the primer sequences of the tested fusion transcripts) RQ–PCR was performed on a 7500-Fast real-time PCR system (Applied Biosystems), using Universal TaqMan Master Mix (2X) (Applied Biosystems), 300 nM of each primer, 100 nM of probe, and 5 *μ*l of cDNA in a total volume of 20 *μ*l. Samples were run in duplicate together with a negative and positive control for each reaction.

## Results

A total of 812 specimens from 762 patients were analysed for *EWSR1* rearrangement by either FISH, RT–PCR or both modalities. After duplicate cases (repeat testing done on the same specimen) were excluded, 772 specimens were included in our analysis (Tables 1, 2, 3). Routine FISH was performed on 753 (97.5%) samples, of which 210 (27.9%) were positive for an *EWSR1* rearrangement and 524 (69.6%) were negative. The FISH study failed in 19 (2.5%) cases. RT–PCR was less commonly used (445 cases, 57.6%). A fusion transcript containing an *EWSR1* rearrangement was documented in 111 (24.9%) samples. Testing failed in 80 (18.0%) cases. Of the 210 FISH-positive cases, RT–PCR was performed in 174, and a fusion transcript was identified in 99/174 (56.9%) cases. Subsequent to a positive FISH result, the initial diagnosis based on morphology and immunohistochemistry was changed in 40 (19.0%) cases (Supplementary Table B). Most of these 40 represented diagnostically complex cases of neoplasms in which there was significant morphologic and immunophenotypic overlap with other tumours of other lineages or other sarcomas. Among these, 14 were initially thought to represent small cell carcinoma and were subsequently changed to a diagnosis of Ewing sarcoma or DSRCT. Other confounding diagnoses included differentiating melanoma from clear cell sarcoma and poorly differentiated synovial sarcoma (which has predominantly round cell morphology) from Ewing sarcoma. In five FISH-negative cases, a fusion transcript was identified and also led to a change in the initial diagnosis. *EWSR1* rearrangement testing (FISH and/or RT–PCR) was performed in 125 undifferentiated neoplasms, of which 62 were composed of spindle cells, 26 of round cells, 7 of pleomorphic cells, with the remainder having mixed morphology. A FISH-positive result was documented in six (4.8%) cases (three spindle, one round and two of mixed morphology). The most common diagnoses for neoplasms that were doubly FISH and RT–PCR negative for *EWSR1-*rearrangements are shown in Supplementary Table C.

### *EWSR1*-rearranged neoplasms

**Figure 1:**
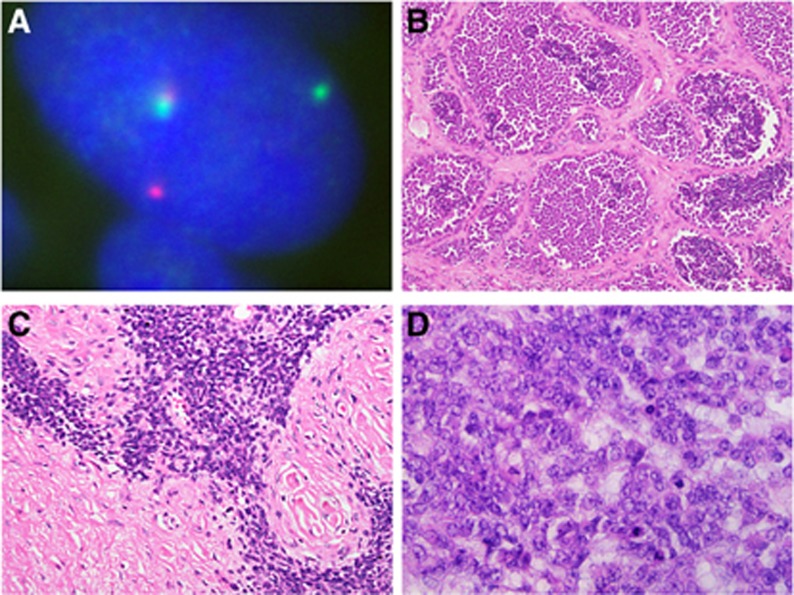
**Fluorescence *in situ* hybridisation for *EWSR1* gene rearrangement, and histology of variant Ewing sarcoma types.** (**A**) Fluorescence *in situ* hybridisation using dual-colour break-apart probes which flank the *EWSR1* breakpoint region on chromosome 22q12. The nucleus of this neoplasm contains separated (split) red and green signals indicating a rearrangement involving the *EWSR1* gene at 22q12. A fused normal signal is also present, denoting the site of the *EWSR1* gene. (**B** and **C**) Ewing sarcoma. These are examples of Ewing sarcomas with morphologic features that can cause diagnostic difficulty, particularly as they can show immunophenotypical overlap with other round cell neoplasms. (**B**) Ewing sarcoma with a well-defined nested architecture and areas of cellular discohesion mimicking alveolar rhabdomyosarcoma; (**C**) Ewing sarcoma with irregular cellular nests in prominent desmoplastic stroma, mimicking desmoplastic small round cell tumour, and (**D**) large cell Ewing sarcoma. RT–PCR in each case was diagnostically contributory, as this showed the presence of *EWSR1-FLI1* fusion transcripts diagnostic of Ewing sarcoma.

Results from FISH and RT–PCR studies routinely used for *EWSR1*-rearranged neoplasms are summarised in Tables 1, 2, 3. The great majority of cases suspected to represent an *EWSR1*-rearranged neoplasm underwent FISH analysis. Suspected cases of Ewing sarcoma, DRSCT, CCS, CCSLGT, PPMS, AFH and EMC were more likely to undergo RT–PCR testing compared to suspected cases of myoepithelial neoplasms and LGFMS. Positive concordance rates between FISH and RT–PCR studies varied between 34.1% and 80.0%. On the basis of morphology and immunohistochemistry, 109 cases were diagnosed as probable or possible Ewing sarcoma. In total, 89 (81.7%) cases had a positive FISH test (*EWSR1* rearrangement) (Figure 1A) and 50 (58.1%) had identifiable *EWSR1-FLI1* or *EWSR1-ERG* fusion transcripts (92.0% and 8.0%, respectively). *EWSR1* fusion transcripts by RT–PCR were not found in 15 (13.8%) FISH-positive cases, with these likely representing rare variant fusions. Furthermore, 18 cases (16.5%) were FISH-negative; 4 (3.7%) had an identifiable fusion transcript and 4 (3.7%) did not; RT–PCR failed or was not performed (due to lack of sufficient material) in the remaining cases. The four cases which were morphologically and immunohistochemically thought to represent Ewing sarcoma but which were FISH and RT–PCR negative are shown in Table 4. All cases comprised hypercellular neoplasms, generally with prominent mitoses and necrosis, and 3/4 were composed of uniform or monotonous small, rounded cells with scanty cytoplasm, with one showing nuclei with moderate or sometimes marked atypia. All showed at least focal expression of CD99 and often of neural/ neuroectodermal markers and were negative for other markers including cytokeratins, desmin, CD34 and haematolymphoid markers. The final interpretations were of small round cell tumour, possibly Ewing sarcoma with variant partner gene, or in the case with cellular atypia, of possible atypical Ewing sarcoma. Clinically, these were all highly aggressive tumours; three of the four patients with follow up died of progressive or metastatic disease within 18 months of diagnosis. The fourth patient had chemotherapy and a below-knee amputation but was subsequently lost to follow up (Table 4). Among the 22 suspected cases of DRSCT, the FISH positivity rate was high (86.3%). RT–PCR reliably identified the *EWSR1-WT1* transcript in 2 of 3 FISH-negative cases. High FISH positivity rates were also documented in CCS (87.9%) and CCSLGT (80.0%). A fusion transcript involving *EWSR1-ATF1* or *EWSR1-CREB1* was identified in all cases of CCSLGT diagnosed pathologically. An *EWSR1* rearrangement was less prevalent in EMC, AFH, PPMS, myoepithelial neoplasms, LGFMS and SEF. As would be typical, no fusion transcript involving *EWSR1-ATF1* or *EWSR1-CREB1* was found in *EWSR*1 FISH-positive myoepithelial neoplasms. Among the *EWSR1*-negative samples, 29 samples were positive for a *FUS* rearrangement either by FISH or RT–PCR: 18 cases were diagnosed as myxoid liposarcoma, 9 cases as LGFMS and 2 cases as SEF.

### Reliability of FISH and RT–PCR

FISH was the more reliable ancillary diagnostic test, with a failure rate for FISH of 2.5% compared with 18.0% for RT–PCR. FISH failure rates remained relatively constant from 2008 through 2015 and were 2.6%, 1.6%, 3.0%, 0.0%, 1.1%, 2.3%, 4.6% and 0.0%, respectively. RT–PCR failure rates were much higher (29.3%, 36.4%, 23.5%, 4.2%, 6.7%, 15.1%, 11.6%, 15.3%, respectively) but did improve over time. FISH was most likely to fail in myoepithelial neoplasms (7.1%) and AFH (5.0%) compared to other *EWSR1*-rearranged neoplasms. The RT–PCR failure rate was highest for Ewing sarcoma (19.8%), DRSCT (20.0%), PPMS (50.0%), LGFMS and SEF (50.0%). Although overall FISH testing was more reliable, the additional RT–PCR testing was useful in identifying a fusion transcript containing an *EWSR1* rearrangement in FISH-negative cases, particularly for Ewing sarcoma (four cases, 3.6%), DRSCT (two cases, 9.1%), AFH (four cases, 20.0%), CCSLGT (one case, 20.0%) and LGFMS and SEF (six cases, 31.6%).

### Unclassifiable *EWSR1*-rearranged neoplasms

The morphologic and immunohistochemical characteristics of the 13 unclassifiable *EWSR1*-rearranged neoplasms are summarised in Table 5. This separate group of cancers was more likely to be diagnosed in an older population (median age 55 years, range 11–82 years). There were no identifiable recurrent morphologic characteristics in this group. Despite extensive immunohistochemical studies coupled with morphologic analysis, three cases were classified as spindle cell sarcomas, not otherwise specified, two as possible myoepithelial neoplasms, one as possible EMC, one as possible solitary fibrous tumour, one as possible myxoid spindle cell sarcoma with adipocytic differentiation and the remainder as unclassifiable neoplasms.

## Discussion

The two main diagnostic platforms available to routine surgical pathology laboratories are FISH and RT–PCR, which are most beneficial when used as complementary modalities. It is now clear though that the spectrum of gene fusions associated with soft tissue tumours is far wider than previously anticipated. Our study exemplifies the widespread nature of *EWSR1* rearrangements in soft tissue tumours and the need to correlate positive molecular findings with morphology and immunohistochemistry. FISH was the more frequently used ancillary test for *EWSR1*-rearranged neoplasms, often because FISH can be performed with scanty amounts of material (using sections cut at 1–4 *μ*m thickness), compared with RT–PCR which initially required sections cut at 20 *μ*m to isolate sufficient RNA (although this is no longer the case, with 3–5 *μ*m sections now routinely used). Another reason for the prevalence of FISH is the lack of available PCR primers to identify rarer fusion variants. Although RT–PCR still has a higher failure rate than FISH, it is a valuable complementary modality, as exemplified in a minority of cases where a fusion transcript was identified while FISH failed or was negative. Plausible explanations for these results include cases where the percentage of neoplastic cells was low and therefore below the threshold where FISH could assign a positive result (i.e., <15% due to a high immune or stromal component) and the presence of rare translocation structures, where the signal configuration could not be easily interpreted as rearranged. While FISH has traditionally been viewed as the more sensitive test compared with RT–PCR, in some tumours such as AFH, RT–PCR shows an equal sensitivity, and may detect cases that are negative with FISH (Thway *et al*, 2015b).

There has been a reduction in the technical failure rates of both modalities over time, but notably of RT–PCR. FISH methods improved with the addition of the extra step described in the methods section. The improved success rate in RT–PCR, including with FFPE material from external institutions, is likely multifactorial. The only protocol change over time (which started around 2011) has been the use of 3–5 *μ*m FFPE sections (rather than 20 *μ*m scrolls) and the deparaffinisation on slides rather than in microcentrifuge tubes. In addition, the greater amount of non-microdissected tissue used previously may have saturated PCR columns with paraffin, undigested material or PCR inhibitor carryover (Vroobel *et al*, 2016). The increasing success in recent years of both RT–PCR and FISH has been markedly aided by the recognition amongst surgical pathology departments of molecular diagnostics (to assess genetic abnormalities in solid neoplasms with available targeted therapies) as a critical component of the patient pathway. This has resulted in widespread improvements from hospitals in fixing and processing pathologic specimens to optimise the tissue available for molecular analysis, with increasing experience of the staff of both surgical pathology and molecular laboratories (Vroobel *et al*, 2016).

Given the increasing spectrum of *EWSR1*-rearranged neoplasms, it is clear that the utility and practicality of ancillary molecular tests need re-evaluation, and it is important that physicians are aware of the limitations of molecular diagnostic techniques, including the non-specificity of many gene fusions. The increasing discovery of characteristic genetic abnormalities in soft tissue neoplasms has caused a significant challenge for molecular diagnostics laboratories in maintaining accredited ancillary tests (van de Rijn *et al*, 2014), compounded by the low cost-benefit ratios due to disease rarity (van de Rijn *et al*, 2014; Mourtzoukou *et al*, 2015). It is important not to disregard the value of immunohistochemistry (Hornick, 2014), which is highly cost-effective and widely available in standard surgical pathology laboratories. Immunohistochemical markers that act as surrogates for molecular investigations are likely to be more cost-effective and rapid. For example, Ewing sarcomas with *EWSR1-ERG* fusions can be detected with the ERG antibody (Wang *et al*, 2012), although this is not specific, as other neoplasms (including vascular tumours, ERG-related myeloproliferative disorders and prostatic adenocarcinomas) express ERG (Miettinen *et al*, 2011).

Although helpful in supporting or confirming diagnoses, FISH and RT–PCR continue to present limitations, and this is exemplified by Ewing sarcomas (Figure 1B–D). The t(11;22)(q24;q12) rearrangement leading to *EWSR1-FLI1* fusion is the commonest (in ∼85% Delattre *et al*, 1992), with about 10% harbouring t(21;22)(q22;q12) leading to *EWSR1-ERG* fusion (Sorensen *et al*, 1994; Maire *et al*, 2008). However, as there are numerous variant fusions (present in less than 1%), ancillary molecular analysis can be negative in tumours displaying typical morphologic and immunohistochemical features of Ewing sarcoma. These variants include the EWSR1-ETS fusions *EWSR1-ETV1* (Jeon *et al*, 1995), *EWSR1-ETV4* (Kaneko *et al*, 1996) and *EWSR1-FEV* (Peter *et al*, 1997) and the TET-ETS fusions *FUS-ERG* (Ichikawa *et al*, 1994; Berg *et al*, 2009) and *FUS-FEV* (Ng *et al*, 2007; Fisher, 2014), as well as rare non-TET/ETS fusions (Mastrangelo *et al*, 2000; Yamaguchi *et al*, 2005; Wang *et al*, 2007; Szuhai *et al*, 2009; Sumegi *et al*, 2011; Fisher, 2014) which are not routinely detected by RT–PCR. Furthermore, a group of primitive round cell tumours with histologic appearances similar to Ewing sarcomas remain unclassifiable, lacking specific clinical and immunohistochemical features and molecular evidence of *EWSR1* gene rearrangements or other small round cell tumour-associated gene rearrangements such as *SS18*, *DDIT3* or *FOXO1*. *FUS* can act as an alternative to *EWSR1* in other neoplasms (Ordonez *et al*, 2009) although this was not routinely requested in the suspected *EWSR1*-rearranged neoplasms assessed, as the role and significance of *FUS* as an alternative to *EWSR1* was not as well known in previous years.

The most common genetic abnormality in small round cell tumours lacking *EWSR1* rearrangement seems to be the *CIC-DUX4* fusion (Italiano *et al*, 2012), in which *CIC* on 19q13.2 fuses with one of the *DUX4* retrogenes on 4q35 or 10q26.3 (Antonescu, 2014). *CIC-DUX4* fusions have been demonstrated in up to two thirds of *EWSR1* rearrangement-negative undifferentiated round cell sarcomas of pediatric and young adult patients (Graham *et al*, 2012; Italiano *et al*, 2012; Antonescu, 2014), with expression profiling demonstrating a distinct gene signature and suggesting a distinct pathogenesis from Ewing sarcoma (Specht *et al*, 2014). *BCOR-CCNB3*-associated round cell neoplasms are also aggressive neoplasms occurring largely in bone or sometimes the deep soft tissues of adolescents or young adults, particularly males (Pierron *et al*, 2012; Puls *et al*, 2014). Although there is a range of histologic appearances, most are composed of undifferentiated small round cells similar to Ewing sarcomas. Immunohistochemically they express nuclear CCNB3 (which is thought to be relatively specific), with most also expressing bcl-2 and approximately two thirds positive for CD99 and CD117 (Pierron *et al*, 2012; Puls *et al*, 2014). Some of the tumours in the small subset of doubly FISH and RT–PCR negative ‘Ewing sarcomas' we identified (Table 4) may possibly represent these newly classified entities.

**Figure 2:**
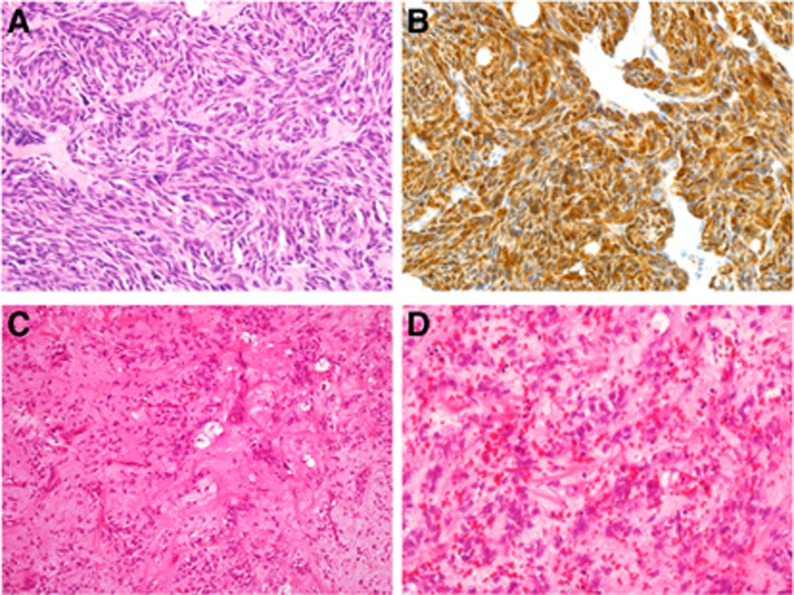
**Examples of neoplasms with *EWSR1* rearrangement but no corresponding detectable *EWSR1*-associated fusion transcripts.** (**A** and **B**) This is a cellular spindle cell neoplasm on the foot of an adult female. Morphologically, this showed nests and sheets of relatively uniform spindle cells (**A**) and was immunohistochemically diffusely positive for S100 protein (**B**). The pathologic features were consistent with clear cell sarcoma (of tendons and aponeuroses), but *EWSR1-CREB1* and *EWSR1-ATF1* fusion transcripts were undetectable with RT–PCR. This may be due to rarer variant translocations or fusion transcripts that are not detectable with commercial primers. (**C** and **D**) This is a neoplasm of sparse to moderate cellularity, composed of patternless distributions of ovoid cells with fibrillary cytoplasm in prominent myxoid stroma with many interspersed thin-walled, medium-sized arcuate vessels. Small numbers of lipoblasts are present (**C**). While the cells are often bland, focally there is some cytologic atypia (**D**), and the features were of a tumour with adipocytic differentiation that was not wholly in keeping with myxoid liposarcoma (MLPS). No *FUS-DDIT3* fusion transcripts (seen in the majority of MLPS) were detectable with RT–PCR. While FISH showed an *EWSR1* rearrangement, no *DDIT3* rearrangement was identifiable (which should be present in myxoid liposarcomas with either *FUS-DDIT3* or *EWSR1-DDIT3* fusions). Therefore this remained an unclassifiable adipocytic neoplasm with myxoid stroma.

Finally, 13 ‘unclassifiable' neoplasms which did not show typical histologic or immunohistochemical features of Ewing sarcoma showed *EWSR1* rearrangement with FISH but no identifiable partner, that is, they did not harbour detectable *EWSR1*-related fusion transcripts with the primers available (Table 5) (Figure 2). Histologically these showed a wide spectrum of architectures and cell types, including spindle, round and ovoid cells, and none of these fitted clearly into any particular group of *EWSR1*-rearranged neoplasms. The new interpretation offered for these was therefore usually of a morphological description such as ‘high grade spindle cell sarcoma.' These tended to be malignant neoplasms often with cellular atypia, necrosis and mitotic figures. None of these had pathologic features typical of *CIC-DUX4*- or *BCOR-CCNB3*-associated sarcomas. It is unclear whether any of these neoplasms could represent specific, as yet uncharacterised tumour types harbouring *EWSR1* rearrangements with unknown partner genes, or if these *EWSR1* rearrangements might be non-reproducible and a function of the intrinsic genetic instability of the tumours. It seems, however, that ‘unclassifiable' *EWSR1*-rearranged neoplasms represent only a small proportion of cases in this study; from these relatively small numbers there were no identifiably recurrent morphologic patterns or immunoprofiles, so the hypothesis of these representing malignant tumours in which *EWSR1* rearrangement was present due to inherent genetic instability rather than as a driver mechanism for pathogenesis appears more likely. Recently, investigators have highlighted four cases of *SMARCB1*-deleted neoplasms of various morphologies (myoepithelial carcinoma, extrarenal rhabdoid tumour, poorly differentiated chordoma and proximal-type epithelioid sarcoma) that also demonstrated *EWSR1* abnormalities with FISH, which had initially led to misinterpretation of these as *EWSR1*-rearranged tumours (Huang *et al*, 2016). FISH had shown heterozygous deletion or unbalanced split signals for *EWSR1,* suggesting unbalanced translocations or rearrangements, but these were in keeping with false-positive results owing to the proximity of the *SMARCB1* and *EWSR1* genes on chromosome 22, whereby large *SMARCB1* deletions can involve the *EWSR1* locus. Care should therefore be taken in the interpretation of FISH for *EWSR1* rearrangement with INI1-deficient neoplasms, and in general the FISH patterns for these tumours have been complex, differing from the more uniform and simple split signals of typical *EWSR1*-rearranged neoplasms (Huang *et al*, 2016).

## Conclusions

This series demonstrates the importance of FISH and RT–PCR as ancillary diagnostic tools in the diagnosis of *EWSR1*-rearranged neoplasms. Although FISH is more sensitive for identifying *EWSR1* rearrangements than RT–PCR, and RT–PCR using commercial primers is limited to only the most prevalent *EWSR1* fusion transcripts, their complementary use is crucial as cases in which FISH is negative may demonstrate *EWSR1*-associated fusion transcripts on RT–PCR. As translocations involving *EWSR1* and its partner genes are often not specific for tumour types and there is significant morphologic and immunohistochemical overlap between groups of *EWSR1-*rearranged neoplasms, it is critical that ancillary molecular findings are always evaluated in specific clinical and pathological context. However, it is also clear that the current routine technologies available in most surgical pathology or molecular diagnostics laboratories (chiefly the two major platforms of FISH and RT–PCR) can only help detect a limited number of fusion genes and these are also low-throughput and labour intensive. The incorporation of high-throughput sequencing technologies into the standard diagnostic repertoire to assess for multiple molecular abnormalities of soft tissue tumours in parallel (including detection of newly characterised Ewing sarcoma-like tumours) might be the most effective and efficient means of ancillary diagnosis in future. This will provide a more definitive classification of many neoplasms that are currently not formally diagnosable with the routine methods available, and enable a specific treatment plan, including entry into appropriate clinical trials for targeted therapies towards specific genetic abnormalities.

## Figures and Tables

**Table 1:** Comparison of FISH and RT–PCR in *EWSR1*-rearranged neoplasms

		**FISH**								**RT–PCR**							
	**Total suspected cases based on histology and IHC**	**Samples tested**		**Positive**		**Negative**		**Failed**		**Samples tested**		**Positive**		**Negative**		**Failed**	
**Histology**	***n***	***n***	**%**	***N***	**%**	***n***	**%**	***n***	**%**	***n***	**%**	***n***	**%**	***n***	**%**	***n***	**%**
Ewing sarcoma	109	109	100.0	89	81.7	18	16.5	2	1.8	86	78.9	50	58.1	19	22.1	17	19.8
DRSCT	22	22	100.0	19	86.3	3	13.6	0	0.0	15	68.2	11	73.3	1	6.7	3	20.0
Myxoid LPS	24	23	95.8	1	4.3	21	91.3	1	4.3	NA	NA	NA	NA	NA	NA	NA	NA
EMC	41	41	100.0	24	58.5	16	39.0	1	2.4	34	82.9	14	41.2	18	52.9	2	5.6
AFH	20	20	100.0	12	60.0	7	35.0	1	5.0	19	95.0	13	68.4	5	26.3	1	5.3
CCS	33	33	100.0	29	87.9	4	12.1	0	0.0	28	84.8	17	60.1	8	28.6	3	10.7
CCSLGT	5	5	100.0	4	80.0	1	20.0	0	0.0	5	100.0	5	100.0	0	0.0	0	0.0
PPMS	2	2	100.0	1	50.0	1	50.0	0	0.0	2	100.0	1	50.0	0	0.0	1	50.0
Myoepithelial neoplasm	42	42	100.0	13	31.0	26	62.0	3	7.1	22	54.4	0	0.0	16	72.7	6	27.3
LGFMS and SEF	19	19	100.0	5	26.3	14	73.7	0	0.0	10	52.6	6	60.0	2	20.0	2	20.0

Abbreviations: AFH=angiomatoid fibrous histiocytoma; CCS=clear cell sarcoma; CCSLGT=clear cell sarcoma-like tumour of the gastrointestinal tract; DSRCT=desmoplastic small round cell tumour; EMC=extraskeletal myxoid chondrosarcoma; IHC=immunohistochemistry; LGFMS=low-grade fibromyxoid sarcoma; LPS=liposarcoma; NA=not applicable; PPMS=primary pulmonary myxoid sarcoma; RT–PCR=reverse transcription–PCR; SEF=sclerosing epithelioid fibrosarcoma.

**Table 2:** Concordance of FISH and RT–PCR positivity according to *EWSR1*-rearranged neoplasm subtype

		**Positive FISH; Positive RT–PCR**		**Positive FISH; Negative RT–PCR**		**Positive FISH; Failed RT–PCR**		**Positive FISH; RT–PCR ND**		**Negative FISH; Positive RT–PCR**		**Negative FISH; Negative RT–PCR**		**Negative FISH; Failed RT–PCR**		**Negative FISH; RT–PCR ND**	
**Histology**	**Samples tested**	***n***	**%**	***n***	**%**	***n***	**%**	***n***	**%**	***n***	**%**	***n***	**%**	***n***	**%**	***n***	**%**
Ewing sarcoma	109	45	41.3	15	13.8	12	11.0	17	15.6	4	3.7	4	3.7	5	4.6	5	4.6
DRSCT	22	9	40.9	0	0.0	3	13.6	7	31.8	2	9.1	1	4.5	0	0.0	0	0.0
Myxoid LPS (FISH and RT–PCR comparison not possible due to lack of commercial *EWSR1-DDIT3* primers)	24	NA	NA	NA	NA	NA	NA	NA	NA	NA	NA	NA	NA	NA	NA	NA	NA
EMC	41	14	34.1	7	17.1	1	2.4	2	4.9	0	0.0	11	26.8	1	2.4	4	9.8
AFH	20	9	45.0	2	10.0	0	0.0	1	5.0	4	20.0	3	15.0	0	0.0	0	0.0
CCS	33	17	51.5	5	15.2	3	9.1	4	12.1	0	0.0	3	9.1	0	0.0	1	0.03
CCSLGT	5	4	80.0	0	0.0	0	0.0	0	0.0	1	20.0	0	0.0	0	0.0	0	0.0
PPMS	2	1	50.0	0	0.0	0	0.0	0	0.0	0	0.0	0	0.0	1	50.0	0	0.0
Myoepithelial neoplasm	42	0	0.0	8	19.0	3	7.1	2	4.8	0	0.0	7	16.7	2	4.8	17	40.5
LGFMS and SEF	19	0	0.0	1	5.3	1	5.3	3	15.8	6	31.6	1	5.3	1	5.3	6	31.6

Abbreviations: AFH=angiomatoid fibrous histiocytoma; CCS=clear cell sarcoma; CCSLGT=clear cell sarcoma-like tumour of the gastrointestinal tract; DSRCT=desmoplastic small round cell tumour; EMC=extraskeletal myxoid chondrosarcoma; LGFMS=low-grade fibromyxoid sarcoma; LPS=liposarcoma; NA=not applicable; ND=not done; PPMS=primary pulmonary myxoid sarcoma; RT–PCR=reverse transcription–PCR; SEF=sclerosing epithelioid fibrosarcoma.

**Table 3:** Results of *EWSR1* and other fusion transcript rearrangements according to histology routinely used at our centre

**Histology**	**Fusion transcript**	***N***
Ewing sarcoma	*EWSR1-FLI1*	46 (92.0%)
	*EWSR1-ERG*	4 (8.0%)
DRSCT	*EWSR1-WT1*	11
EMC	*EWSR1-NR4A3*	13 (81.3%)
	*TAF15-NR4A3*	3 (18.8%)
AFH	*EWSR1-ATF1*	3 (23.0%)
	*EWSR1-CREB1*	10 (77.0%)
CCS	*EWSR1-ATF1*	16 (94.1%)
	*EWSR1-CREB1*	1 (5.9%)
CCSLGT	*EWSR1-ATF1*	2 (40.0%)
	*EWSR1-CREB1*	3 (60.0%)
PPMS	*EWSR1-CREB1*	1

Abbreviations: AFH=angiomatoid fibrous histiocytoma; CCS=clear cell sarcoma; CCSLGT=clear cell sarcoma-like tumour of the gastrointestinal tract; DSRCT=desmoplastic small round cell tumour; EMC=extraskeletal myxoid chondrosarcoma; PPMS=primary pulmonary myxoid sarcoma.

**Table 4:** Pathological characteristics of the four cases which were pathologically thought to represent Ewing sarcoma, but which were negative for both *EWSR1* rearrangement with FISH and for *EWSR1-FLI1* and *EWSR1-ERG* fusion transcripts with RT–PCR

**Case no.**	**Age/sex/site**	**Clinical features/follow up**	**Pathology**
1	40/F/buttock	15 cm buttock primary with inguinal metastases. Resected. Metastases to inguinal nodes after 9 months; chemotherapy. Resection and adjuvant radiotherapy. Died of progressive disease 1 year after diagnosis	Extensively necrotic, cystic and haemorrhagic cellular tumour of moderately pleomorphic ovoid cells with vesicular nuclei, prominent nucleoli and scanty cytoplasm. Frequent mitoses and apoptoses. Focal CD99+ all other markers negative, including CD56, desmin, neurofilament, S100 protein, AE1/AE3, EMA, CD30, CD45 and TdT Final interpretation: ‘Undifferentiated malignant neoplasm, possibly atypical Ewing sarcoma'
2	23/M/pelvis	Presented with irritative bladder symptoms and back pain. Imaging: 15 cm solid mass in posterior pelvis in presacral space. Chemotherapy and radical radiotherapy. Developed pulmonary metastases and died of disease 18 months after diagnosis	Partially necrotic cellular tumour of sheets of uniform round cells with rounded nuclei, prominent nucleoli and scanty, sometimes clear cytoplasm. Focally moderate to strong positivity for CD99, with focal CD56 and focal nuclear S100 protein. All other markers negative, including desmin, SMA, AE1/AE3, EMA, CD34, NB84, chromogranin, synaptophysin, NSE, CD30, CD45, CD138 and TdT Final interpretation: ‘Small round cell tumour. Possibly Ewing sarcoma with variant partner gene'
3	13/F/leg	3-year history of bilateral leg and foot discomfort. Imaging: soft tissue mass dorsal to the talus and anterior to ankle joint. Chemotherapy. Left below-knee amputation after 6 months	Cellular tumour composed of sheets of uniform small cells with round to oval nuclei without atypia, and focally clear cytoplasm and occasional macronucleoli. Mitotic index of 4/10 hpf; no necrosis. Areas of adjacent fibrosis but no osteoid or chondroid present. Diffuse strong positivity for CD99 and NSE. All other markers negative, including TLE1, CD56, desmin, SMA, AE1/AE3, EMA, CD34, chromogranin, synaptophysin, CD45 and TdT Final interpretation: ‘Small round cell tumour. Possibly Ewing sarcoma with variant partner gene. Findings not in keeping with small cell osteosarcoma or mesenchymal chondrosarcoma'
4	22/F/cerebellum	Left cerebellar mass. Resected. Regrowth after 1 month; debulking, chemotherapy and radiotherapy. Further debulking after 7 months. Chemotherapy but died of disease 10 months after diagnosis	Cellular malignant neoplasm composed of small to medium-sized relatively uniform cells with round nuclei, fine dispersed chromatin, occasional nucleoli and scanty cytoplasm, with some cytoplasmic clearing. Focal necrosis, as well as mitotic figures and apoptoses. Focal CD99, FLI1 and EMA+ INI1+ desmin, GFAP, chromogranin, synaptophysin, NB84 and ERG− Final interpretation: ‘Small round cell tumour. Possibly Ewing sarcoma with variant partner gene'

Abbreviations: F=female; hpf = high power fields; M=male; RT–PCR=reverse transcription–PCR.

**Table 5:** Pathological characteristics of the 13 unclassifiable cases with positive *EWSR1* rearrangement on FISH

**Case no.**	**Age/sex/site**	**Pathology**
1	33/M/shoulder	Fascicular spindle to focally more polygonal cell tumour; moderate to marked atypia. Extensive necrosis and mitoses. CD34+ in most cells; focal nuclear S100 protein+, very focal AE1/AE3, CD99 and EMA Final interpretation: ‘Sarcoma NOS'
2	47/F/foot	Multinodular spindle and polygonal cell tumour. Focal rhabdoid features and hemangiopericytic pattern. bcl-2, INI1 and focally CD99+, very focal S100 protein+ Final interpretation: ‘Findings not conclusive. Spindle cell sarcoma NOS; possible myoepithelial tumour, MPNST or extraskeletal myxoid chondrosarcoma'
3	52/M/shoulder	Small ovoid cells separated by fibrous septa. Largely solid; focal myxoid change. Focal necrosis and prominent mitoses. Focal EMA; all other markers negative. Features inconclusive for myoepithelial carcinoma. Some areas resembling EMC Final interpretation: ‘Malignant neoplasm, unclassifiable'
4	56/M/shoulder	Spindle and ovoid cell neoplasm, focal myxoid change, microcyst formation. Focal CD34, bcl-2 and CD99+ Final interpretation: ‘Findings not conclusive. Some MLPS-like features. Possibly solitary fibrous tumour'
5	11/M/subcutis of forearm	Moderately circumscribed lesion of plump ovoid and spindle cells with elongated cytoplasmic processes in myxoid stroma. No significant pleomorphism or necrosis. EMA, INI1 and focal D2-40+ claudin-1, cytokeratin and CD34− Final interpretation: Features resembling epithelioid perineurioma but immunophenotype not wholly supportive. Possible EMC
6	54/M/kidney	Fascicular spindle cell tumour; moderate atypia; focal necrosis and prominent mitoses. Focal nuclear S100 protein, AE1/AE3, CD99, CD56 and CD34+ Final interpretation: ‘High-grade spindle cell sarcoma'
7	27/M/oropharynx	Epithelioid cells with mild atypia in nests in hyalinised or basement membrane-like stroma; basaloid cells also present. Diffuse strong MNF116, EMA, CK5/6, CK14 and p63+. CD10 and calponin+in peripheral cells Final interpretation: ‘Probable myoepithelial neoplasm without identifiable partner gene'
8	57/F/neck	Small to medium-sized epithelioid and polygonal cells with spindle cell areas. Up to one mitotic figure in 50 high power fields. Possible foci of adipocytes. Diffuse nuclear S100 protein and p16+ focal desmin and GFAP+. INI1+ Final interpretation: ‘Borderline tumour which cannot be conclusively characterised'
9	69/M/thyroid	Polygonal and spindle cell neoplasm. Focal variable FLI1, INI1, TLE1, CD56, CD99 and bcl-2+ Final interpretation: ‘Polygonal and spindle cell tumour of indeterminate (probably mesenchymal) lineage, but not otherwise classifiable. Does not fit into any specific class of *EWSR1*-rearranged neoplasm'
10	82/M/neck	Focally necrotic polygonal cell tumour. S100 protein, synaptophysin and weak focal EMA and chromogranin+ No detectable *EWSR1-CREB1* or *EWSR1-ATF1* fusions with RT–PCR. No *BRAF* mutation detected
11	64/F/not available	Focally infiltrative biphasic neoplasm with bland spindle cells and epithelioid component, with occasional mitoses but no necrosis. Epithelioid component positive for CK7 and EMA Final interpretation: ‘Possible myoepithelial neoplasm'
12	34/M/spinal cord	Infiltrative tumour composed of ovoid cells in irregular cords within fibrous tissue. Focally EMA+, S100 protein, STAT6 and MUC4− Final interpretation: ‘Benign mesenchymal neoplasm of uncertain lineage. Possibly myoepithelial tumour but immunophenotype incomplete'
13	60/F/thigh	Myxoid spindle cell tumour; prominent myxoid stroma. Areas of adipocytic differentiation (but morphology not in keeping with myxoid liposarcoma). Occasional mitotic figures; no necrosis. Diffuse S100 protein and p16+ weak focal CDK4+. No *DDIT3* rearrangement with FISH Final interpretation: ‘Myxoid spindle cell sarcoma with adipocytic differentiation'

Abbreviations: DSRCT=desmoplastic small round cell tumour; EMC=extraskeletal myxoid chondrosarcoma; F=female; M=male; MLPS=myxoid liposarcoma; MPNST=malignant peripheral nerve sheath tumour; RT–PCR=reverse transcription–PCR.

NB: Full immunopanels (including pancytokeratins, S100 protein, CD34, desmin and SMA) were performed for each case. Only positive findings are listed in most cases.
