# Supplementary material for: The spectrum of EWSR1-rearranged neoplasms at a tertiary sarcoma centre; assessing 772 tumour specimens and the value of current ancillary molecular diagnostic modalities
Source: Br J Cancer. 2017 Jan 31;116(5):669–78. doi: 10.1038/bjc.2017.4 (PMC5344299; doi:10.1038/bjc.2017.4)
Supplement: Supplementary Tables [file bjc20174x1.docx]

**Supplementary Material**

**Supplementary Table A: Primer sequences**

| **Fusion transcript/gene** | **Primer sequence** | **Reference** |
| --- | --- | --- |
| ***B2M*** | B2M-246Fw TGACTTTGTCACAGCCCAAGATA | Hostein *et al*, 2004 |
|  | B2M-230Rv AAT CCA AAT GCG GCA TCT TC |  |
|  | B2M-P TGATGCTGCTTACATGTCTCGATCCCA |  |
| ***EWSR1-ERG*** | EWSR1 x7F TCCTACAGCCAAGCTCCAAGTC | Jin *et al*, 2003 |
|  | ERG x6R GGTTGAGCAGCTTTCGACTG |  |
| ***EWSR1-FLI1*** | EWSR1-FLI1-Fw CCAAGTCAATATAGCCAACAG | Lewis *et al*, 2007 |
|  | EWSR1-FLI1-Rv GGCCAGAATTCATGTTATTGC |  |
|  | EWSR1/FLI1(P1) ACGGGCAGCAGAACCCTTCTTAT |  |
|  | EWSR1/FLI1(P2) ACGGGCAGCAGAGTTCACTGCT |  |
| ***EWSR1-WT1*** | EWSR1 x7F TCCTACAGCCAAGCTCCAAGTC | Jin *et al*, 2003 |
|  | WT1 x8R ACCTTCGTTCACAGTCCTTG |  |
| ***EWSR1-NR4A3(I)*** | EWSR1-NR4A3_I_Fw GCGATGCCACAGTGTCCTATG | Okamoto *et al*, 2001 |
|  | EWSR1-NR4A3_I_Rv ATATTGGGCTTGGACGCAGGG |  |
| ***EWSR1-NR4A3(II)*** | EWSR1-NR4A3_II_Fw CTCCAAGTCAATATAAGCCAAC |  |
|  | EWSR1-NR4A3_II_Rv GGACGTCCGGCGAGGCGAAGC |  |
| ***EWSR1-NR4A3(III)*** | EWSR1-NR4A3_III_Fw TCTGGCAGACTTCTTTAAGCA |  |
|  | EWSR1-NR4A3_III_Rv GGACGTCCGGCGAGGCGAAGC |  |
| ***EWSR1-ATF1(1)*** | EWSR1 8Fw CATGAGCAGAGGTGGGCG | Coindre *et al*, 2006 |
|  | ATF1 4Rv CCCCGTGTATCTTCAGAAGATAAGTC |  |
|  | ATF1 probe AGGAGGACGCGGTGGAATGGG |  |
| ***EWSR1-ATF1(2)*** | EWSR1 7Fw GCCAAGCTCCAAGTCAATATAGC |  |
|  | ATF1 5Rv CAACTGTAAGGCTCCATTTGGG |  |
|  | ATF2 probe CAGAGCAGCAGCTACGGGCAGCA |  |
| ***EWSR1-CREB1x8*** | EWSR1 Ex7-Fw TCCTACAGCCAAGCTCCAAGTC | Antonescu *et al*, 2002; Antonescu *et al*, 2007. |
|  | CREB1 Ex8-Rv GGTATGTTTGTACGTCTCCAGAGG |  |
| ***EWSR1-CREB1x7*** | EWSR1 Ex7-Fw TCCTACAGCCAAGCTCCAAGTC |  |
|  | CREB1 Ex7-Rv GTACCCCATCGGTACCATTGT |  |

**Supplementary Table B. Tumours with diagnostic change following the discovery of an *EWSR1* rearrangement**

| **Case number** | **Initial diagnosis based on morphology and immunohistochemistry** | **Final diagnosis following the finding of a positive *EWSR1* rearrangement** |
| --- | --- | --- |
| 1 | Myoepithelioma | EMC |
| 2 | Undifferentiated carcinoma | Ewing sarcoma |
| 3 | Rhabdomyosarcoma | Ewing |
| 4 | Melanoma | CCS |
| 5 | Malignant neoplasm NOS | EMC |
| 6 | Poorly differentiated neural/ neuroendocrine tumour | Ewing sarcoma |
| 7 | Possibly small cell carcinoma | DRSCT |
| 8 | Tumor with neuroendocrine differentiation, possibly small cell carcinoma | Ewing sarcoma |
| 9 | Primary pulmonary myxoid sarcoma | EMC |
| 10 | MPNST | EMC |
| 11 | Rhabdomyosarcoma | DRSCT |
| 12 | Myxoid spindle cell sarcoma | EMC |
| 13 | Malignant spindle and polygonal cell tumour suggestive of melanoma | CCS |
| 14 | Poorly differentiated synovial sarcoma | Ewing sarcoma |
| 15 | Nerve sheath tumour, cellular schwannoma | CCSLGT |
| 16 | Malignant tumor without specific differentiation. Possibly myxoid chondrosarcoma | CCS |
| 17 | Myoepithelioma | Angiomatoid fibrous histiocytoma |
| 18 | Neuroendocrine carcinoma | DRSCT |
| 19 | Glomus tumour | Ewing sarcoma |
| 20 | Small round cell tumour, possibly DSRCT | Ewing sarcoma |
| 21 | Small round cell tumour consistent with although not typical of Ewing sarcoma | Myoepithelial carcinoma |
| 22 | Melanoma vs MPNST | CCSLGT |
| 23 | Melanoma | CCS |
| 24 | Carcinoma (no specific differentiation or site of origin noted) | Myoepithelial carcinoma |
| 25 | Synovial sarcoma | Ewing sarcoma |
| 26 | Benign or low-grade spindle cell lesion of mesenchymal lineage | Angiomatoid fibrous histiocytoma |
| 27 | Undifferentiated malignant neoplasm, probably sarcoma | Myoepithelioma |
| 28 | Digital fibromyxoma | Myoepithelioma |
| 29 | Small cell carcinoma | Ewing sarcoma |
| 30 | CD99-positive small round cell tumour | Ewing sarcoma |
| 31 | Malignant rhabdoid tumour | Myoepithelial carcinoma |
| 32 | Spindle cell tumour without obvious lineage | EMC |
| 33 | Neuroendocrine carcinoma | Ewing sarcoma |
| 34 | MPNST vs melanoma, vs CCS | Ewing sarcoma |
| 35 | Small cell carcinoma | Ewing sarcoma |
| 36 | Poorly differentiated biphasic synovial sarcoma | Ewing sarcoma |
| 37 | Clear cell odontogenic carcinoma | CCS |
| 38 | Spindle cell neoplasm of uncertain lineage | Angiomatoid fibrous histiocytoma. |
| 39 | Small cell carcinoma | Ewing sarcoma |
| 40 | Poorly differentiated synovial sarcoma | Ewing sarcoma |

**Abbreviations:** CCS clear cell sarcoma, CCSLGT clear cell sarcoma-like tumour of the gastrointestinal tract, DSRCT desmoplastic small round cell tumour, EMC extraskeletal myxoid chondrosarcoma, MPNST malignant peripheral nerve sheath tumour, NOS not otherwise specified.

**Supplementary Table C. Most commonly retained diagnoses for *EWSR1*-negative samples**

| **Diagnosis** | **Number of cases** |
| --- | --- |
| Melanoma | 43 |
| Well-differentiated/dedifferentiated liposarcoma | 36 |
| Carcinoma | 36 |
| Synovial sarcoma | 11 |
| Epithelioid and rhabdoid tumors | 7 |
| Rhabdomyosarcoma | 12 |
| Myxoid liposarcoma | 23 |
